# Supplementary material for: G-protein coupled receptor 35 (GPR35) regulates the colonic epithelial cell response to enterotoxigenic Bacteroides fragilis
Source: Commun Biol. 2021 May 14;4:585. doi: 10.1038/s42003-021-02014-3 (PMC8121840; doi:10.1038/s42003-021-02014-3)
Supplement: Supplementary file 2 — Supplementary Information [file 42003_2021_2014_MOESM2_ESM.pdf]

Supplementary Information

G-protein coupled receptor 35 (GPR35) regulates the colonic epithelial cell response to enterotoxigenic Bacteroides fragilis

Annemarie Boleij[1,2*], Payam Fathi[1], William Dalton[3], Ben Park[3*], Xinqun Wu[1], David Huso[4**], Jawara Allen[1,] Sepideh Besharati[5], Robert A. Anders[5], Franck Housseau[6], Amanda E. Mackenzie[7], Laura Jenkins[7], Graeme. Milligan[7], Shaoguang Wu[1, #], Cynthia L. Sears[1, #]

Supplementary Figures

Supplementary figure 1

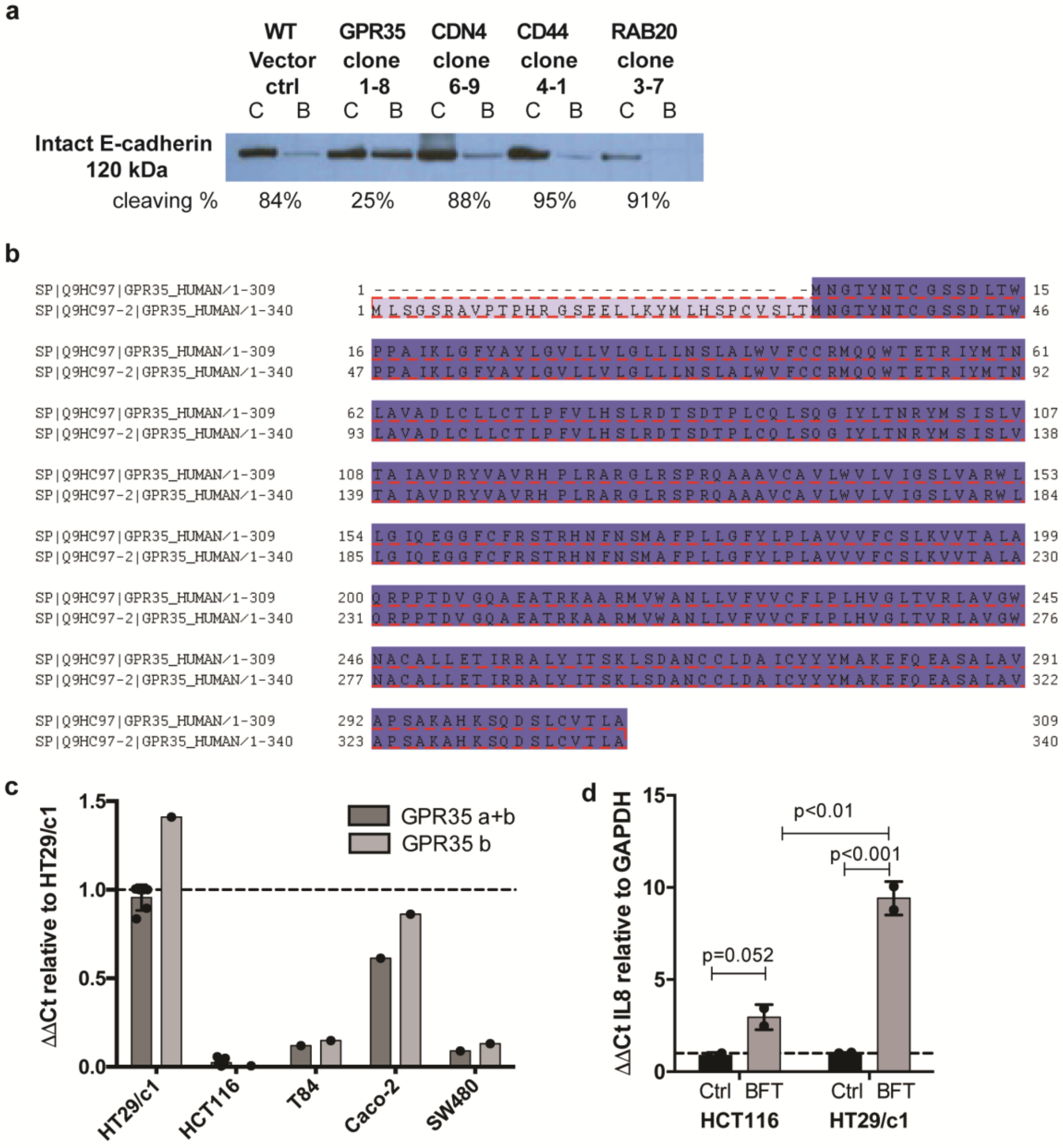

Supplementary Figure 1: A) GPR35 shRNA knock-down inhibited E-cadherin cleavage in response to BFT in HT29/c1 cells (25% in clone 1-8 (GPR35) vs 84% in the vector control (WT)), shRNA knock-down of Claudin 4 (CDN4), CD44 and RAB20 had no effect on E-cadherin cleavage by BFT. B) amino acid sequence of GPR35 a (309 amino acids) and b (340 amino acids). GPR35 b is 31 amino acids longer at the N-terminal side of the protein. C) mRNA expression of the long GPR35 isoform (GPR35b) or both the short and long isoform (GPR35 a+b) in HT29/c1 (N=7), HCT116 (N=7), SW480 (N=1), Caco-2(N=1) and T84 (N=1) CRC epithelial cell lines.

Data are presented relative to HT29/c1 cells and corrected for GAPDH expression (ΔΔCt HT29/c1). HT29/c1 cells show the highest expression of GPR35 and HCT116 the lowest. GPR35b is the main expressed isoform in all cell lines. D) mRNA expression of IL8 in HT29/c1 and HCT116 cells exposed to BFT for 4 hours is 9.46-fold and 3.34-fold higher than non-treated control cells (N=2).

Supplementary figure 2

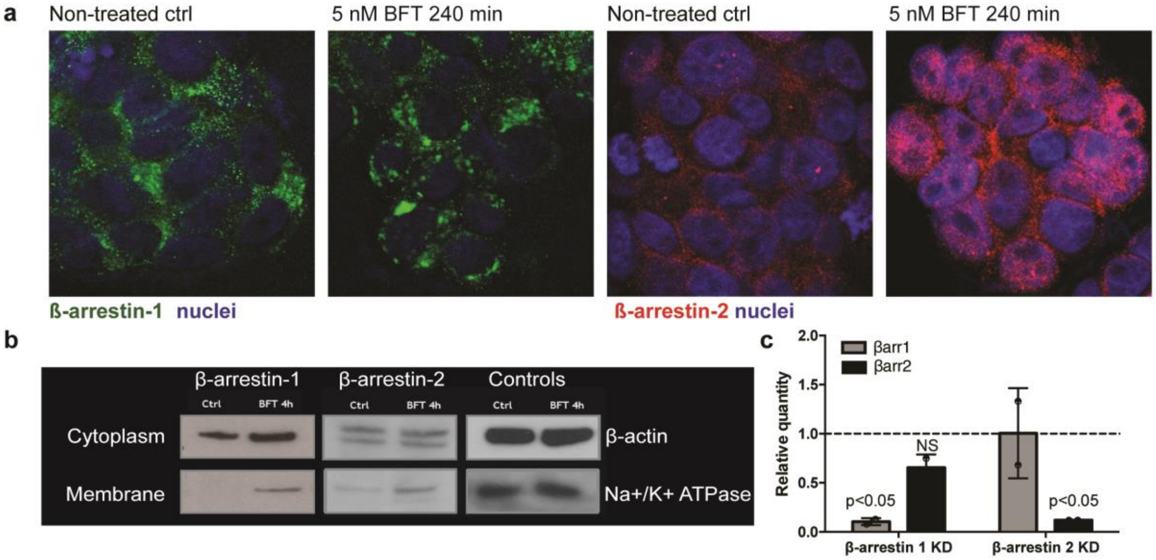

Supplementary Figure 2: A) 2D confocal images extracted from middle of z-stack. Note the more diffuse staining of β-arr1 (green) and β-arr2 (red) in the non-treated controls compared to a different distribution and more punctual staining in BFT-treated cells. B) The β-arr1 and β-arr2 signal are increased upon BFT-treatment for 4 hours (240 min) especially in the membrane fraction (representative figure, 20.5 fold and 1.79 fold respectively, N=2 and 3 respectively). C) creation of β-arrestin1 and 2 KD cells resulted in significant reduction of mRNA expression of β-arrestin-1 ($p<0.05$) and β-arrestin-2 ($p<0.05$) respectively.

Supplementary figure 3

a

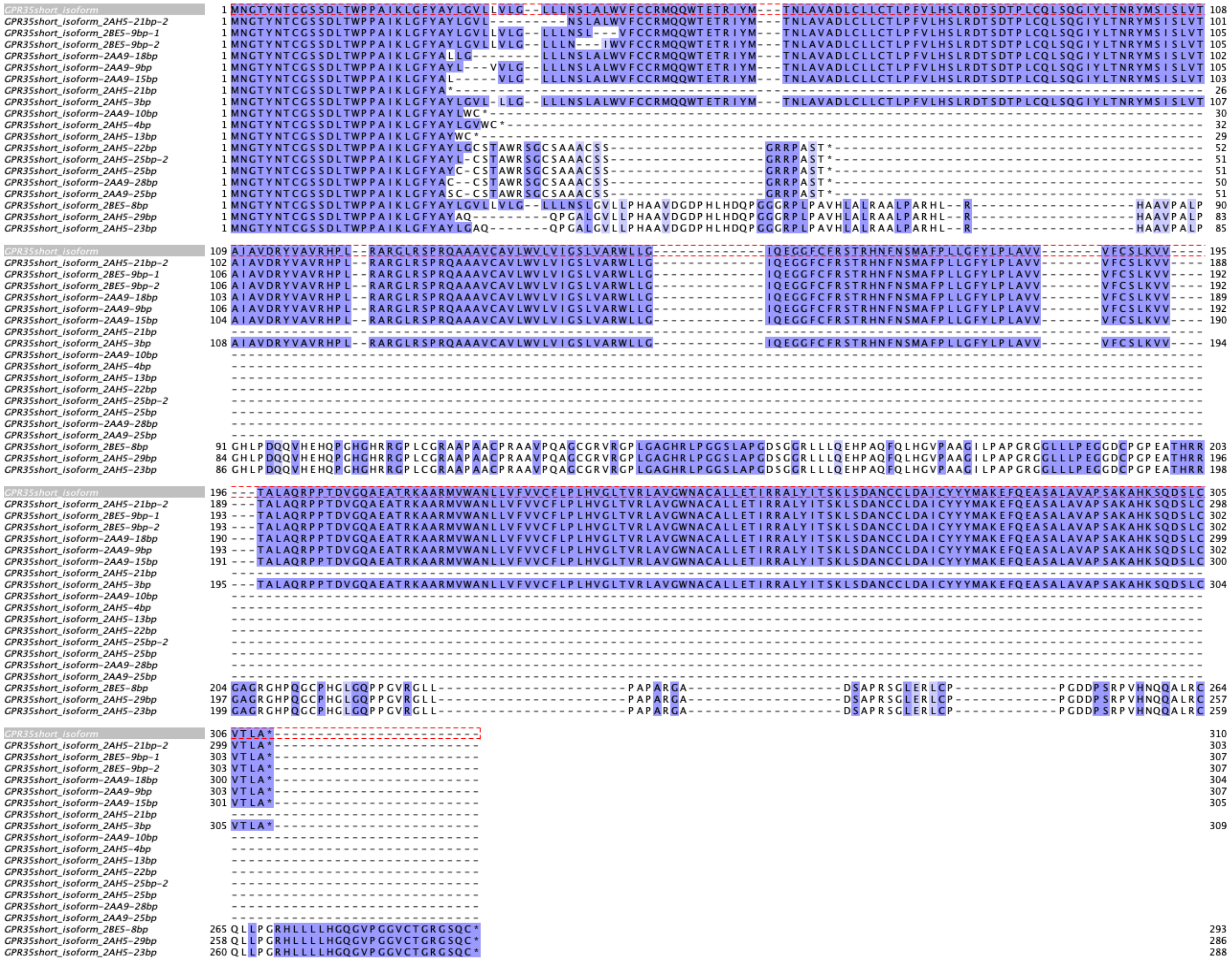

b

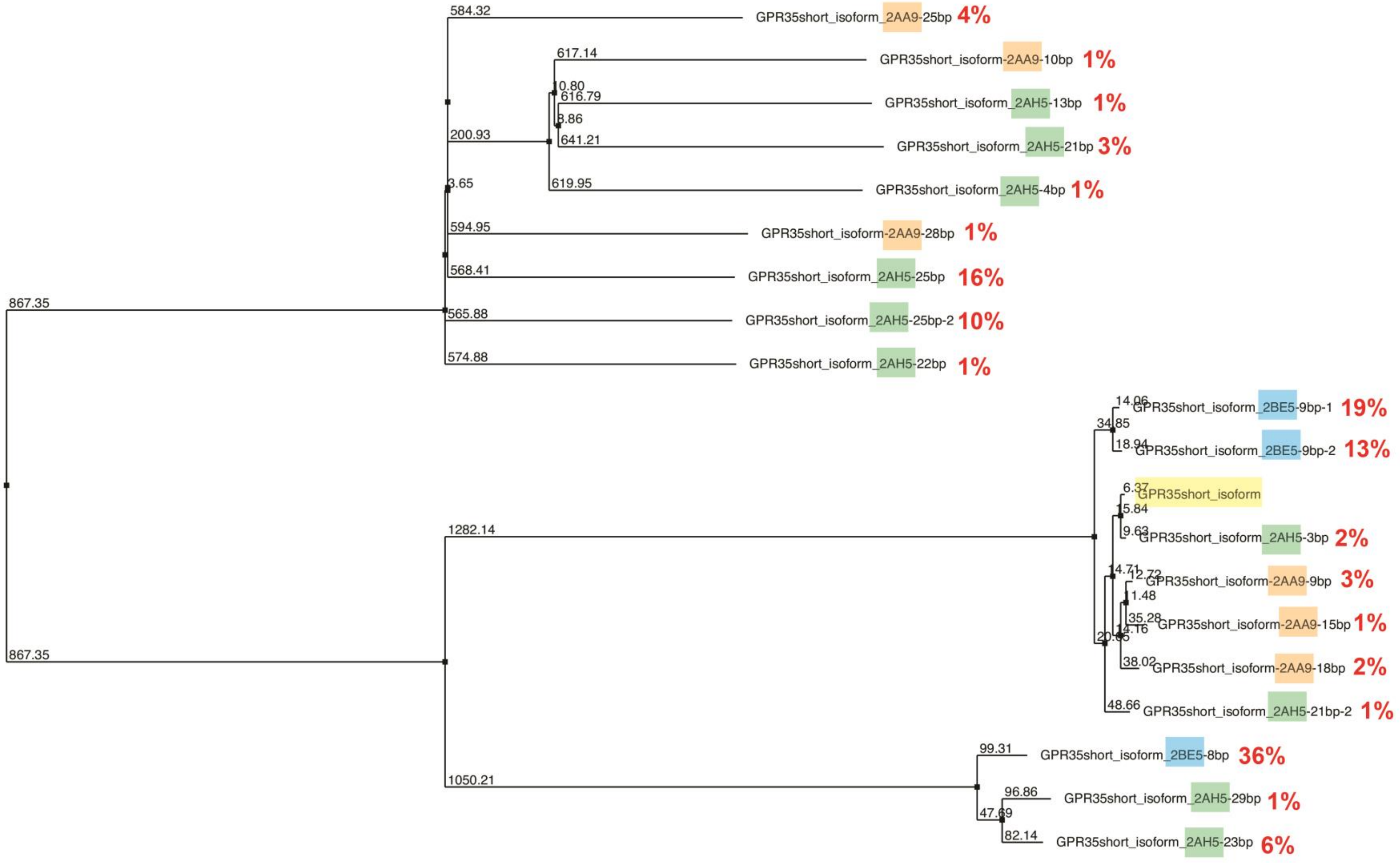

Supplementary figure 3: A) Protein alignment with CLUSTALW Omega of the short isoforms of human WT GPR35 and all CRISPR-CAS KO clones. All in frame deletions show close resemblance to WT GPR35 while the frameshift deletions of 2AH5 (10/12 – 39% of total), 2AA9 (3/6 – 6% of total) and 2BE5 (1/3 -36% of total) are clearly different in composition. Similar amino acids are colored in blue. B) A phylogenetic tree based on a point mutation matrix (PAM) of the suggested protein translations of short isoforms of WT GPR35 (yellow) and the GPR35 CRISPR-CAS KO clones 2AH5 (green), 2BE5 (blue) and 2AA9 (orange). The in frame deletions of 2BE5 (32%), 2AH5 (3%) and 2AA9 (6%) show significant similarity to WT GPR35. In red the percentage of sequences contributing to the sequence pools of the clones.

Supplementary figure 4

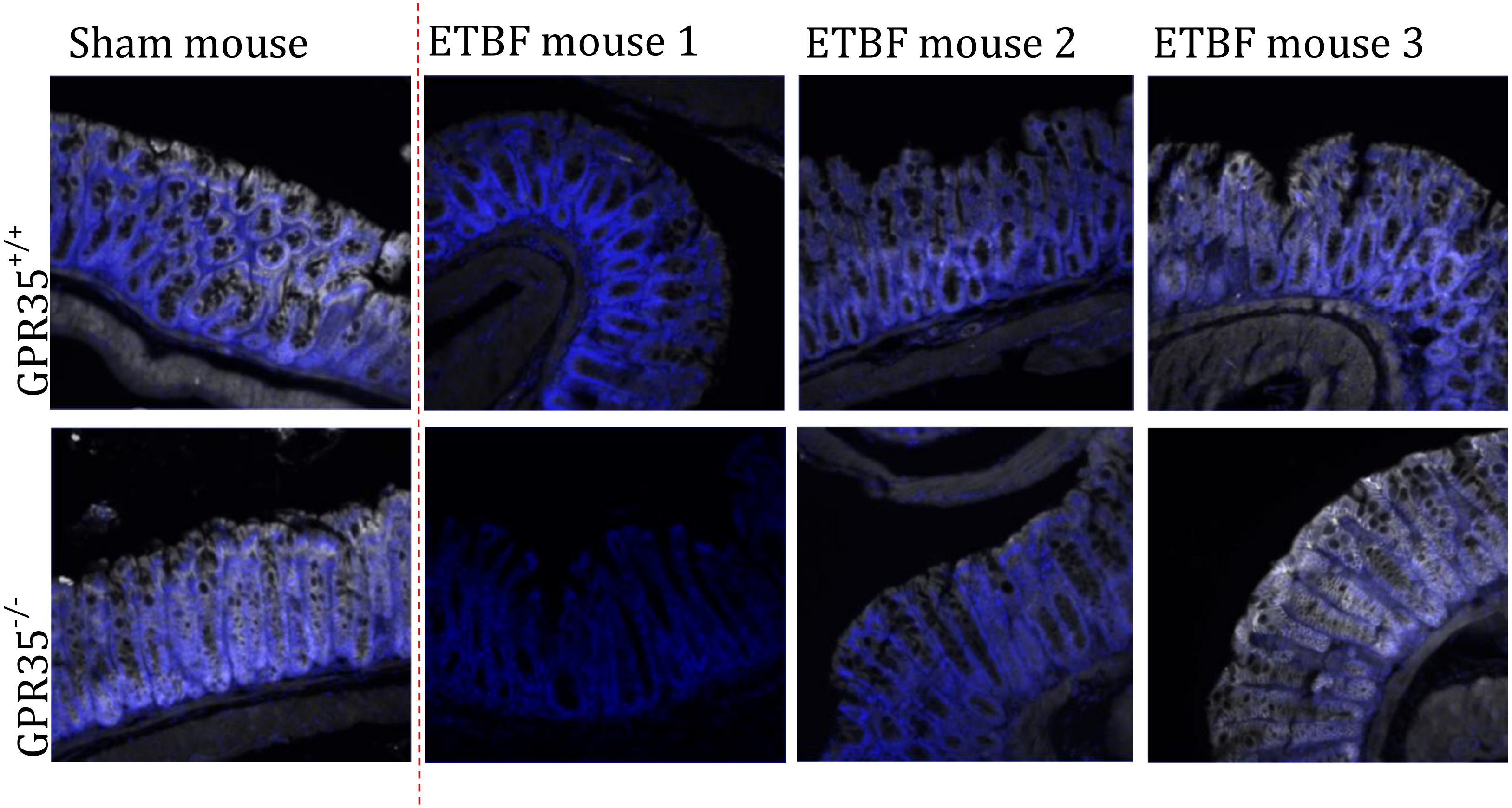

Supplementary figure 4: E-cadherin staining of GPR35$^{+/+}$ and GPR35$^{-/-}$ mice colonized with ETBF for 3 days. Representative pictures of 3 ETBF-colonized mice are shown compared to the sham control. E-cadherin staining is similarly reduced in ETBF mouse 1 and 2 GPR35$^{+/+}$ or GPR35$^{-/-}$; in mouse 3 E-cadherin staining is shown in a region with largely intact E-cadherin in GPR35$^{+/+}$ and GPR35$^{-/-}$ mice. No clear difference was observed between GPR35-proficient and -deficient mice. E-cadherin staining (white) and nuclear stain DAPI (blue).

Supplementary figure 5

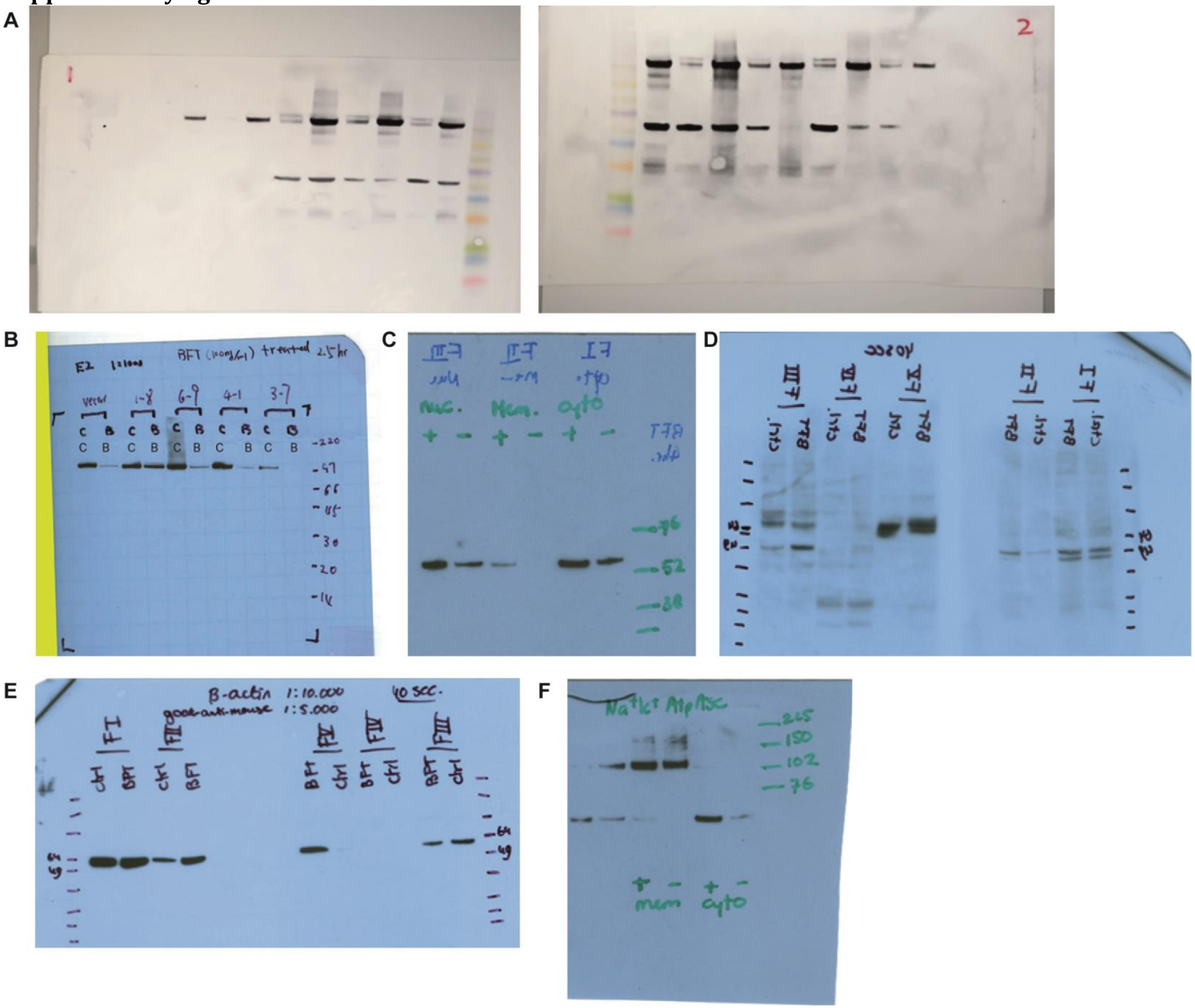

Supplementary figure 5: A) *E-cadherin and actin Western blot from figure 4d*. E-cadherin at ~120 kDa and actin at ~42 kDa. Short exposure time in blot 1 for KO2AH5 and KO2AA9 and long exposure time 2 for KO2BE5. From right to left: lane 1:wildtype HT29/c1 (ctrl),lane 2: wildtype HT29/c1 (BFT), lane 3: KO 2AH5 (ctrl), lane 4: KO2AH5 (BFT),lane 5: KO2AA9 (ctrl), lane 6: KO2AA9 (BFT), lane 7: KO 2BE5 (ctrl), lane 8: KO 2BE5 (BFT). The top bands at ~120 kDa show E-cadherin and the bottom bands beta-actin primed on the same blot. Rainbowmarker is shown on the right. B) *E-cadherin western blot from supplementary figure 1a*: E-cadherin at ~120 kDa. From left to right: lane 1: vector (ctrl), lane 2: vector (BFT), lane 3: GPR35 clone 1-8 (ctrl), lane 4: GPR35 clone 1-8 (BFT) lane 5: CDN4 clone 6-9 (ctrl), lane 6: CDN4 clone 6-9 (BFT),lane 7: CD44 clone 4-1 (ctrl),lane 8: CD44 clone 4-1 (BFT), lane 9: RAB20 clone 3-7 (ctrl), lane 10:RAB20 clone 3-7 (BFT) Size ladder is noted in black. C) *Beta-arrestin 1 Western blot from supplementary figure 2b* : Beta-arrestin 1 at ~50 kDa. From right to left: lane 1: cytoplasm FI (ctrl), lane 2: cytoplasm FI (BFT), lane 3: Membrane FII (ctrl), lane 4: membrane FII (BFT), lane 5: nucleus FIII (ctrl), lane 6: nucleus FIII (BFT). Size ladder is noted in green. D) *Beta-arrestin 2 Western blot from supplementary figure 2b*. Beta-arrestin 2 at ~50 kDa. From left to right: lane 1: cytoplasm FI (ctrl), lane 2: cytoplasm FI (BFT), lane 3: Membrane FII (ctrl), lane 4: membrane FII (BFT), lane 5: cyto-skeleton FV (BFT), lane 6: cyto-skeleton FV (ctrl), lane 7: chromatin bound FIV (BFT), lane 8: chromatin bound FIV (ctrl), lane 9: nucleus FIII (BFT), lane 10: nucleus FIII (ctrl). Size ladder is noted in black. E) *Beta-actin Western blot from supplementary figure 2b*. From left to right: lane 1: cytoplasm FI (ctrl), lane 2: cytoplasm FI (BFT), lane 3: Membrane FII (ctrl), lane 4: membrane FII (BFT), lane 5: cyto-skeleton FV (BFT), lane 6: cyto-skeleton FV (ctrl), lane 7: chromatin bound FIV (BFT), lane 8: chromatin bound FIV (ctrl), lane 9: nucleus FIII (BFT), lane 10: nucleus FIII (ctrl). Size ladder is noted in black . F) *Na+K+ ATPase Western blot from supplementary figure 2b*. Na+K+ ATPase at ~100 kDa. From right to left: lane 1: cytoplasm FI (ctrl), lane 2: cytoplasm FI (BFT), lane 3: Membrane FII (ctrl), lane 4: membrane FII (BFT) Size ladder is noted in green.

Supplementary Tables

Supplementary Table 1

ID	Systematic	Common	Genbank	BROAD Instritute TRC#	Cell shape change on 1st KD cells	Cell shape change on 2nd KD cells	cell shape change tested on stable KD clones	UniPro ID	Gene Title_Affymetrix	96 well plate ID
**12**	201428_at	CLDN4	NM_001305	TRCN0000116627	neg or reduced (N=2)	reduced (N=1)	pos or reduced (N=2)	O14493	claudin 4	6-9
**27**	210264_at	GPR35	AF089087	TRCN0000008887	reduced (N=1)	reduced (N=1)	neg or reduced (N=5)	Q9HC97	G protein-coupled receptor 35	1-8
**55**	219622_at	RAB20	NM_017817	TRCN0000048093	pos or reduced (N=2)	reduced (N=1)	reduced (N=1)	Q9NX57	Ras-related protein Rab-20	3-7
