# Supplementary material for: G-protein coupled receptor 35 (GPR35) regulates the colonic epithelial cell response to enterotoxigenic Bacteroides fragilis
Source: Commun Biol. 2021 May 14;4:585. doi: 10.1038/s42003-021-02014-3 (PMC8121840; doi:10.1038/s42003-021-02014-3)
Supplement: Supplementary file 3 — Description of Additional Supplementary Files [file 42003_2021_2014_MOESM3_ESM.pdf]

Description of Additional Supplementary Files

File name: Supplementary Data 1

Description: List of 82 genes screened with shRNA KD for effect on BFT signaling in HT29/C1 cells. For each shRNA tested, the corresponding common gene names, Genbank IDs, Broad institute TRC number, validation of the KD (1st and 2nd time), the UniProt ID and full gene title in the Affymetrix database are shown.

File name: Supplementary Data 2

Description: Source data used to create the figures in the main manuscript. Each tab contains the data belonging to the corresponding figure.
