# Supplementary material for: G-protein coupled receptor 35 (GPR35) regulates the colonic epithelial cell response to enterotoxigenic Bacteroides fragilis
Source: Commun Biol. 2021 May 14;4:585. doi: 10.1038/s42003-021-02014-3 (PMC8121840; doi:10.1038/s42003-021-02014-3)
Supplement: Supplementary file 6 — Reporting Summary [file 42003_2021_2014_MOESM6_ESM.pdf]

nature research

Corresponding author(s): Annemarie Boleij

Last updated by author(s): Mar 3, 2021

Reporting Summary

Nature Research wishes to improve the reproducibility of the work that we publish. This form provides structure for consistency and transparency in reporting. For further information on Nature Research policies, see our Editorial Policies and the Editorial Policy Checklist.

Statistics

For all statistical analyses, confirm that the following items are present in the figure legend, table legend, main text, or Methods section.

n/a	Confirmed	
☐	☒	The exact sample size (*n*) for each experimental group/condition, given as a discrete number and unit of measurement
☐	☒	A statement on whether measurements were taken from distinct samples or whether the same sample was measured repeatedly
☐	☒	The statistical test(s) used AND whether they are one- or two-sided<br>*Only common tests should be described solely by name; describe more complex techniques in the Methods section.*
☒	☐	A description of all covariates tested
☒	☐	A description of any assumptions or corrections, such as tests of normality and adjustment for multiple comparisons
☐	☒	A full description of the statistical parameters including central tendency (e.g. means) or other basic estimates (e.g. regression coefficient) AND variation (e.g. standard deviation) or associated estimates of uncertainty (e.g. confidence intervals)
☐	☒	For null hypothesis testing, the test statistic (e.g. F, t, r) with confidence intervals, effect sizes, degrees of freedom and P value noted<br>*Give P values as exact values whenever suitable.*
☒	☐	For Bayesian analysis, information on the choice of priors and Markov chain Monte Carlo settings
☒	☐	For hierarchical and complex designs, identification of the appropriate level for tests and full reporting of outcomes
☒	☐	Estimates of effect sizes (e.g. Cohen's d, Pearson's r), indicating how they were calculated

Our web collection on statistics for biologists contains articles on many of the points above.

Software and code

Policy information about availability of computer code

Data collection	no software was used
Data analysis	JALview version 2.11.1.0 (www.jalview.org)

For manuscripts utilizing custom algorithms or software that are central to the research but not yet described in published literature, software must be made available to editors and reviewers. We strongly encourage code deposition in a community repository (e.g. GitHub). See the Nature Research guidelines for submitting code & software for further information.

Data

Policy information about availability of data

All manuscripts must include a data availability statement. This statement should provide the following information, where applicable:

- Accession codes, unique identifiers, or web links for publicly available datasets
- A list of figures that have associated raw data
- A description of any restrictions on data availability

All data is available within the manuscript and Supplemental files. Material requests and correspondence about data can be addressed to Annemarie.Boleij@radboudumc.nl.

Field-specific reporting

Life sciences study design

All studies must disclose on these points even when the disclosure is negative.

Sample size	no sample size calculations were performed for the mice experiments. For the experiments with the GPR35 KO mice the sample size per experiment were dependent on the littermates. We performed the mice experiments at least 3 times for each time point resulting in n=6+ per group from three independent experiments.<br><br>For the in vitro experiments, the number of replicates are noted in each graph. each experiment was repeated at least once. The majority of the experiments were repeated 3 times.
Data exclusions	No data were excluded from the in vitro analysis. For the mice experiments one sham mice was excluded that was cross-infected with an unknown pathogen.
Replication	All findings reported were reproduced and are represented in the graphs. (see sample size)
Randomization	experimental wells for in vitro cell experiments and mice in cages were randomly placed in groups, making sure there was no batch effect of experimental plates in vitro and cage effects for mice.
Blinding	researchers were blinded for scoring toxin acitvity. The other in vitro experiments were not blinded. The pathology data from the mice experiments were scored by a pathologist that was blinded for the experimental conditions.

Reporting for specific materials, systems and methods

We require information from authors about some types of materials, experimental systems and methods used in many studies. Here, indicate whether each material, system or method listed is relevant to your study. If you are not sure if a list item applies to your research, read the appropriate section before selecting a response.

Materials & experimental systems

n/a	Involved in the study
☐	☒ Antibodies
☐	☒ Eukaryotic cell lines
☒	☐ Palaeontology and archaeology
☐	☒ Animals and other organisms
☒	☐ Human research participants
☒	☐ Clinical data
☒	☐ Dual use research of concern

Methods

n/a	Involved in the study
☒	☐ ChIP-seq
☒	☐ Flow cytometry
☒	☐ MRI-based neuroimaging

Antibodies

Antibodies used	Primary antibodies:<br>mouse-anti-beta-arrestin 1 (BD, 610551),<br>Rabbit-anti-beta-arrestin 1 (Abcam, ab32099)<br>Goat anti-beta-arrestin 2 (Abcam, ab31294),<br>Rabbit anti-Na+/K+ ATPase (Thermo Scientific, PA5-17251)<br>mouse anti HDAC2 (3F3), (Cell signaling, #5113)<br>Mouse anti-beta-actin (Sigma, A5441),<br>Rabbit anti-E-cadherin (E2),(Gift from James Nelson, standford University specific for the 25 amino acids of the cytoplasmic domain of E-cadherin),<br>Rabbit anti-GFP [E385](Abcam, ab32146),<br>Mouse anti-FLAG M2 (Sigma Aldrich, F3165)<br><br>Secondary antibodies:<br>Donkey-anti-goat-alexa fluor 568 (Fisher scientific, #A-11057),<br>Goat-anti-rabbit-alexa fluor 488 (ThermoFisher scientific, #A-27034),<br>Donkey anti-goat HRP (Promega, V0851),<br>goat-anti rabbit HRP (Jackson, 111-035-144),<br>goat anti-mouse HRP (Jackson, 115-035-003)
Validation	Mouse-anti-beta-arrestin 1 (https://www.bdbiosciences.com/us/reagents/research/antibodies-buffers/cell-biology-reagents/cell-biology-antibodies/purified-mouse-anti--arrestin-10beta-arrestin1/p/610551),<br>goat-anti-beta-arrestin 2 (https://www.abcam.com/beta-arrestin-2-antibody-ab31294.html),<br>Rabbit anti-Na+/K+ ATPase, validation info no longer available on Thermo Scientific website, product is discontinued.<br>mouse anti HDAC2, (https://www.cellsignal.com/products/primary-antibodies/hdac2-3f3-mouse-mab/5113?

Ntk=Products&Ntt=5113)
Mouse-anti-beta-actin (https://www.sigmaaldrich.com/catalog/product/sigma/a5441?lang=en&region=NL),
Rabbit-anti-GFP (https://www.abcam.com/gfp-antibody-e385-ab32146.html),
Mouse-anti-FLAG (https://www.sigmaaldrich.com/catalog/product/sigma/f3165?lang=en&region=NL&gclid=Cj0KCQjwqfz6BRD8ARIsAIXQCf2PcdButee29m_QZb5_qlKHLPpZoG-thLzximdKpBv4poOVAC8gkvsaAsWlEALw_wcB)

Eukaryotic cell lines

Policy information about cell lines

Cell line source(s)	HT29/c1 is a cloned HT29 cell, obtained from Daniel Louvard from Intistute Pasteur. The cells are derived from a human colon carcinoma. HEK293T cells with Flp-In T-rex were provided by Graeme milligan.
Authentication	The HT29/c1 cell line was obtained in 1992 and selected based on BFT sensitivity (Weikel et al. Infect Immin 1992). The cell line has been extensively studied in its response to BFT over the past 2 decades and original stocks are stored in liquid nitrogen.
Mycoplasma contamination	The stocks in the liquid nitrogen were tested negative for mycoplasma contamination. we did not test for mycoplasma contaminatination for each experiment.
Commonly misidentified lines (See ICLAC register)	Not applicable

Animals and other organisms

Policy information about studies involving animals; ARRIVE guidelines recommended for reporting animal research

Laboratory animals	mouse, C57bl6, wild-type and GPR35 KO (Gpr35tm1(KOMP)Vlcg) male and female.
Wild animals	the study did not involve wild animals.
Field-collected samples	the study did not involve samples collected from the field.
Ethics oversight	The mouse protocols were approved by the Johns Hopkins University Animal Care and Use Committee in accordance with the Association for Assessment and Accreditation of Laboratory Animal Care International.

Note that full information on the approval of the study protocol must also be provided in the manuscript.
